# Flight After Stapes Surgery: An Evidence‐Based Recommendation

**DOI:** 10.1002/oto2.65

**Published:** 2023-07-19

**Authors:** Taim Akhal, Marc Bassim

**Affiliations:** ^1^ School of Medicine, Royal College of Surgeons in Ireland Medical University of Bahrain Busaiteen Bahrain; ^2^ Department of Otolaryngology Cleveland Clinic Abu Dhabi UAE

**Keywords:** airplane, auditory tube, aviation, ear surgery, eustachian tube, flight, flying, middle ear, otology, pressure, stapes surgery

## Abstract

**Objective:**

Recommendations for air travel after stapes surgery, specifically stapes surgery, vary, with no standard recommendation to guide patients and surgeons. According to our search, no previous article has explored the physics of middle ear changes during flight and its effects on poststapedectomy patients in a systematic way. The aim of this study is to bring together 2 arms of expertise, otology, and aviation, to produce an evidence‐based recommendation for flight after stapes surgery.

**Data Sources:**

The database MEDLINE was searched during August 2022. The search strategy had the goal of identifying studies that discovered the effects of flying on stapes surgery patients and the effects of atmospheric pressure on middle ear structures.

**Review Methods:**

The articles yielded from the search strategy were transferred to the online citation manager Rayyan. Included in the review were those studies reporting patient outcomes after flying following ear surgery; additional studies included those reporting pressure changes in the middle ear and ossicular chain displacement whether in experimental or animal conditions.

**Conclusion:**

Modern‐day commercial air travel is safe for patients who have undergone stapedotomy surgery, even very shortly after hospital discharge if they have to.

**Implications for Practice:**

If *stapedotomy* patients wish to fly after hospital discharge, otologists are to reassure them that it is safe to do so. Patients are to be reminded to perform a gentle Valsava maneuver about every 4 minutes during airplane descent.

There is significant heterogeneity among practicing surgeons regarding air travel recommendations after stapes surgery. In 1996, Harrill et al^1^ performed a survey of 231 physician members of the American Otological Society and the American Neurotology Society (AOS and ANS) regarding their recommendations for travel, snorkeling, and scuba diving after different ear surgeries. They noticed no consensus regarding physicians' practice patterns in this regard, with recommendations ranging from no restriction (12% of respondents) to 4‐ to 6‐month restriction; 60% of physicians recommended a 2‐week abstinence, while 20% recommended no more than a 2‐day restriction. Interestingly, the duration of air travel restriction was inversely related to the physician's experience; 63% physicians with <100 stapedotomies recommended a >2‐week restriction, whereas only 49% of physicians that performed >100 stapedotomies recommended more than a 2‐week restriction.

Similarly, a national survey of 134 British otologists was done in 2016, investigating common stapes surgery practice: the most common recommendation regarding flying was to wait at least 6 weeks after stapes surgery.^2^

The above recommendations were not reported to be based on any specific science, but more on empiric surgeon experience and patterns of practice. We, therefore, aim to review the science analyzing pressure changes during flights in modern commercial aircraft, its reported effect on middle ear volume and pressure changes, and the resultant ossicular chain displacement. We hypothesize that postoperative flight poses insignificant risk on stapes surgery patients.

## Rationale

The main concern regarding flying after stapes surgery relates to the development of a perilymphatic fistula at the stapedotomy/stapedectomy side, with a secondary concern being prosthesis displacement, either into the vestibule or out of position.

The mechanism of perilymphatic fistula formation relates to the displacement of the oval window seal and a resultant perilymph fluid leak, causing acute vestibulopathy and sensorineural hearing loss. The concern is that outward displacement of the piston due to volume changes in the middle ear, will pull the unhealed tissue seal away from the footplate fenestration. Alternatively, inward displacement of the piston toward the vestibule may result in damage to the membranous labyrinth, specifically the saccule, with temporary or permanent hearing loss and dizziness.

The piston displacements described above would result from the significant barometric pressure changes, especially during ascent and descent, with the resultant volume change and tympanic membrane displacement. These mechanisms, as we will describe below, are dependent on the rate of pressure changes in the cabin, and the ability of the eustachian tube to offset such changes, minimizing the effect on the middle ear.

## Methods

**Figure 1:**
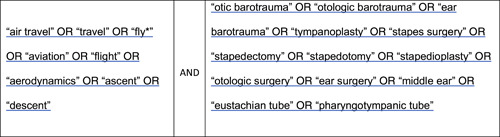
MEDLINE search strategy. This figure outlines the search terms that were input onto PubMed to yield our results.

**Figure 2:**
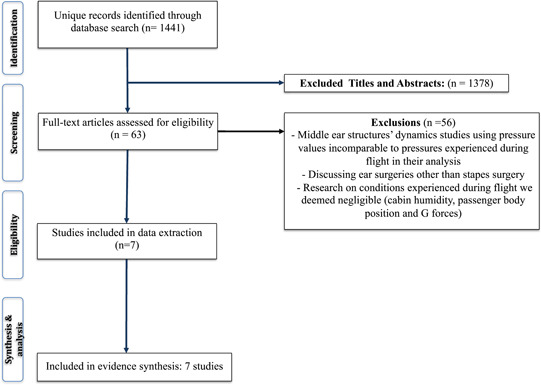
Preferred reporting items for systematic reviews and meta‐analyses diagram. This figure showcases the process of study elimination throughout the screening period of this paper.

A comprehensive search strategy was utilized to search the database MEDLINE during August 2022 (Figure 1). The search strategy was constructed to identify studies reporting the effects of flying on stapes surgery patients and the effects of atmospheric pressure on middle ear structures. The yielded results were transferred to the online screening software Rayyan. After title and abstract screening, 63 full‐text papers qualified for further review (Figure 2). Middle ear structures' dynamics studies using pressure values comparable to those experienced during the commercial flight in their analysis, or studies showcasing recommendations for stapes surgery patients, were included. Some reasons for study exclusion were middle ear structures' dynamics studies using pressure values incomparable to pressures experienced during the commercial flight in their analysis, discussing ear surgeries other than stapes surgery, and research on conditions experienced during flight that are not of significant importance for commercial passengers (eg, G force effect, more relevant for military personnel).

## Part I—The Physics

### Modern Aircraft and Pressure Changes

In 1998, a study investigated 16 US Air force crew members who returned to flight after stapedectomy surgery. The study discussed a protocol for evaluating aircrew members after surgery to return to flight and, based on their review of a variety of cases in their archive dating to before the guidelines era, recommended a grounding duration of 3‐ to 6 months postop provided that all testing is normal.^3^ The more recent guidelines of the US Navy, US Air Force, and National Aeronautics and Space Administration (NASA), however, require a 3‐month grounding period with the absence of any hearing loss or vertigo in the postop period after a stapedectomy.^4^ Military aircrew, however, are subjected to very rapid altitude changes and significant accelerations, not typically experienced in commercial flights.

### Pressure Changes and Eustachian Tube Function

The Federal Aviation Association (FAA) states that the cabin pressure altitude (pressure inside the cabin) should not exceed 8000 ft at the maximum operating altitude in a normally functioning airplane.^5^ Cabin rate (rate of pressure change in the cabin) varies based on a number of factors like flight conditions and commercial owners' choices. The ascent and descent cabin rate of modern aircrafts typically ranges from 300 to 500 ft/min.^6^ Aircrafts like the Boeing 737 and the Airbus A320 have a maximum *descent* cabin rate of 750 ft/min.^7^, ^8^ We will be making our comparisons based on the maximum cabin altitude proposed by the FAA and the maximum cabin rate during descent of 750 ft/min.

During ascent, the ambient air pressure decreases gradually, and the middle ear is filled with relatively positive‐pressure air compared to the environment. In patients with healthy eustachian tube function, the eustachian tube passively opens at a pressure differential of 15 mmHg, and vents out the positive‐pressure air.^9^ This process of passive venting, therefore, occurs on average every 400 ft of ascent and is rarely a problem.^9^ During descent, however, the ambient pressure increases as the aircraft gets closer to sea level. The pressure in the middle ear is now relatively negative, and the action of the eustachian tube opening is now an active process. Muscular activity evoked via swallowing, yawning or Valsalva's maneuver is usually sufficient to open the auditory tube.

If, however, the pressure differential between the ear and nasopharynx (or ambient air) becomes more than 80 mmHg, the nasopharyngeal end of the eustachian tube is now closed at a force greater than that which can be developed by the muscles that open the tube,^9^ resulting in progressively increased negative pressure in the middle ear and eventual barotrauma.

The change in cabin altitude in pressure units during descent is not linear; in Table 1, we calculated pressure changes for every 750 ft of descent at 15°C. Rates vary between 17 and 20 mmHg, with the changes in pressure increasing as the flight gets closer to sea level. By instructing the patient to actively open their eustachian tube every 3‐ to 4 min of descent, the middle ear pressures are equalized regularly without much force, avoiding any significant change in volume and associated prosthesis displacement. These 17 to 20 mmHg differentials are well within the opening ranges of the eustachian tube, and significantly below the threshold of 80 mmHg. If, however, the pressure is not equalized, the buildup is cumulative, potentially reaching the 80 mmHg threshold. As noted above, there is no need for any instruction for ascent or cruising cabin altitude as the eustachian tube will *passively* open at every 15 mmHg of pressure decrease.

**Table 1:** Pressure Changes Throughout Flight Descent

Cabin altitude (m) (ft)	Pressure (mmHg)	Change in pressure from the previous 228.6 m (750 ft) (mmHg)
2438.4 (8000)	564	N/A
2209.8 (7250)	581	17
1981.2 (6500)	598	17
1752.6 (5750)	615	17
1524 (5000)	632	17
1295.4 (4250)	650	18
1066.8 (3500)	669	19
838.2 (2750)	687	18
609.6 (2000)	707	20
381 (1250)	726	19
152.4 (500)	746	20
0 (sea level)	760	14 (note that this change was over 152.4 m or 500 ft)

Column 1 presents the cabin altitude values every 750 ft in descending order. Column 2 presents the corresponding atmospheric pressure value, in mmHg, that is experienced at that altitude. Column 3 presents the change in pressure experienced by the passenger for every 750 ft of descent.

Even though there is a limited number of reports of patients flying after stapes surgery, no evidence of barotrauma was found in a report of scuba divers and skydivers, who are subjected to a much higher pressure change^10^; the limitation of this report, however, is that the time after surgery was defined as a range of 3 to 60 months, a period that may have given enough time for the tissue seal to complete its healing.

## Part II—The Biology

### Pressure Changes and Piston Displacement

Several studies have reported on prosthesis displacement when the ear is subjected to varying pressures. Jiang and his team utilized cadaveric temporal bones to investigate stapes footplate (SFP) motion under blast exposure. They subjected the ear at the entrance of the ear canal to 48 kPa of pressure (the equivalent of 360 mmHg change).^11^ Using Laser Doppler Vibrometry (LDV), they estimated the mean displacement of the SFP under these conditions to be around 68.7 μm.

Hüttenbrink evaluated the effects of ambient pressure changes on piston prosthesis displacement in a cadaveric study. Based on his findings and measurements, he recommended that flying be permitted approximately 2 weeks after a stapedioplasty, a period where the oval window seal is allowed to heal.^12^

Another experiment by Hüttenbrink exposed 9 fresh temporal bone middle ears to ±400 mmH_2_O (±29 mmHg) pressures; these bones had a piston prosthesis implanted through stapedioplasty. With these ranges of pressure, the pistons displaced an average of 232 μm in either direction, with a maximum displacement noted at 500 μm in certain bones. Furthermore, they noted that piston displacement plateaus as the pressure was increased, probably due to limitation on movement of the malleus/incus complex.

The pressures used in the above studies are well above the average change of 15 mmHg experienced during ascent, or the 20 mmHg every 750 ft during descent. Based on the above, it would be logical to assume that a stapes piston would be unlikely to displace more than 0.5 mm in extreme cases.

The membrane of the saccule is usually at an average of 0.8 mm medial to the undersurface of the footplate. In stapedotomy cases, the recommended insertion depth of the prosthesis is 0.25 mm deeper to the footplate. Even with the maximal displacements described above, this would still be unlikely to cause any damage to the vestibule. On the other hand, assuming an average footplate thickness of 0.25 mm, an outward displacement of the piston of 0.5 mm would still be unlikely to result in displacement out of the fenestration and the formation of a fistula.

## Stapedotomy Versus Stapedectomy

By nature of the procedure, stapedectomy results in a larger fenestration into the SFP, necessitating soft tissue coverage and increasing the risk of perilymphatic fistula formation. Most of the previous data dictating recommendations come from the era of stapedectomy, with the period of restriction on flight pertaining to allowing this soft tissue seal to heal. Stapedotomy, on the other hand, requires a smaller fenestration with the piston itself acting as a seal, even if no soft tissue is used.

The 1996 AOS and ANS survey by Harril did not reveal increased baroprotection with stapedotomies as opposed to stapedectomies, although this might be due to the relatively lower number of stapedotomies investigated; they did, however, describe that 69% of reported cases of perilymphatic fistulae occurred when a tissue seal was used^1^, suggesting a potentially higher risk in stapedectomy patients.

## Implications for Practice

Granted that a *stapedotomy* patient wishes to fly soon after surgery, the available literature suggests that it is safe to do so in a commercial setting. Patients are to be reminded to perform gentle Valsalva maneuver about every 4 minutes during airplane descent. Otologists are to teach stapes surgery patients how to perform a gentle, yet effective, Valsalva maneuver; this is to avoid an overzealous action that poses piston displacement risks. If there is any concern for eustachian tube dysfunction, prescribing pseudoephedrine 120 mg 30 minutes prior to flight appears to significantly reduce the risk of barotrauma.^13^

For *stapedectomy* patients, we recommend a 2‐week restriction from flight where the tissue seal is allowed to heal to ensure sufficient frictional resistance, in line with the results by Huttenbrink et al.

These guidelines are important for patient counseling and scheduling of surgery, especially for practices who treat patients referred from distant geographical locations.

Note that these data do not necessarily apply to patients who underwent other types of ear surgery (eg, tympanoplasty), as the mechanics would be different in these cases. Future research should focus on reporting on patient outcomes after air travel following ear surgery to support the extant literature and the current recommendations.

## Conclusion

The common recommendation for patients to wait 3 months after stapes surgery before flying is not supported by the current literature. Indeed, as demonstrated by the different studies summarized here, and based on recent aviation data, the expected pressure changes during flights are unlikely to result in the formation of a perilymphatic fistula or the displacement of the piston. Based on the above data and reviews, there should be no time restriction on flights after stapedotomy surgery, provided the patient has a normally functioning Eustachian tube; they should be reminded to perform a gentle Valsalva maneuver about every 4 minutes on descent, to minimize pressure changes. Flights beyond 2 weeks after stapedectomy surgery should be deemed safe, as the tissue seal over the oval window is expected to have healed by then.

## Limitations

Some of the data reported in this review is taken from cadaveric studies, which may not faithfully reproduce conditions in living patients. Similarly, the number of patients who have been reported in the literature to fly shortly after stapes surgery is limited, though several surgeons, as reported above have minimal restrictions on flight as per their own report.

## Author Contributions

**Taim Akhal**, conducting, editing, and presenting the research; **Mark Bassim**, study design, analysis, and editing.

## Disclosures

### Competing interests

None.

### Funding source

None.

## References

[1] Harrill WC , Jenkins HA , Coker NJ . Barotrauma after stapes surgery: a survey of recommended restrictions and clinical experiences. Am J Otol. 1996;17(6):835‐845. 8915410

[2] Lancer H , Manickavasagam J , Zaman A , Lancer J . Stapes surgery: a National Survey of British Otologists. Eur Arch Otrhinolaryngol. 2016;273(2):371‐379. 10.1007/s00405-015-3560-6 25711736

[3] Thiringer JK , Arriaga MA . Stapedectomy in military aircrew. Otolaryngol Head Neck Surg. 1998;118(1):9‐14. 10.1016/S0194-5998(98)70368-7 9450822

[4] Rajguru R . Post stapedotomy aviation: A changing scenario. Indian J Occup Environ Med. 2014;18(3):105‐108. 10.4103/0019-5278.146905 25598613 PMC4292193

[5] 14 CFR § 25.841—Pressurized cabins . LII/Legal Information Institute. Accessed August 3, 2022. https://www.law.cornell.edu/cfr/text/14/25.841

[6] Control of cabin pressure—aircraft pressurization systems (Part 4). Aircraft Systems . Accessed August 3, 2022. https://www.aircraftsystemstech.com/2017/05/control-of-aircraft-cabin-pressure.html#:~:text=In%20addition%20to%20the%20modes

[7] Brady C . The Boeing 737 pressurisation system. The Boeing 737 Technical Site. Accessed August 3, 2022. http://www.b737.org.uk/pressurisation.htm#:~:text=In%20all%20737%27s%20the%20pr%20essurisation

[8] hursts.org.uk.‌4. Pressurisation . Accessed August 3, 2022. https://hursts.org.uk/airbus-technical/html/ar01s04.html

[9] Mirza S , Richardson H . Otic barotrauma from air travel. J Laryngol Otol. 2005;119(5):366‐370. 10.1258/0022215053945723 15949100

[10] House JW , Toh EH , Perez A . Diving after stapedectomy: clinical experience and recommendations. Otolaryngol Head Neck Surg. 2001;125(4):356‐360. 10.1067/mhn.2001.118183 11593171

[11] Jiang S , Dai C , Gan RZ . Dual‐laser measurement of human stapes footplate motion under blast exposure. Hear Res. 2021;403:108177. 10.1016/j.heares.2021.10817 33524791

[12] Hüttenbrink KB . Clinical significance of stapedioplasty biomechanics: swimming, diving, flying after stapes surgery. Adv Otorhinolaryngol. 2007;65:146‐149. 10.1159/000098791 17245036

[13] Jones JS , Sheffield W , White LJ , Bloom MA . A double‐blind comparison between oral pseudoephedrine and topical oxymetazoline in the prevention of barotrauma during air travel. Am J Emerg Med. 1998;16(3):262‐264. 10.1016/s0735-6757(98)90097-3 9596428

